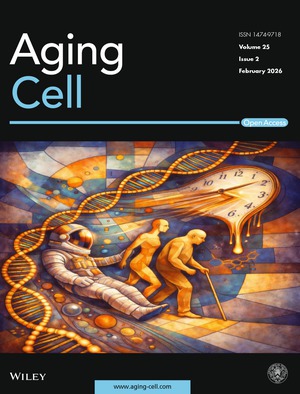# Featured Cover

**DOI:** 10.1111/acel.70415

**Published:** 2026-02-15

**Authors:** Matías Fuentealba, JangKeun Kim, Jeremy Wain Hirschberg, Bader Shirah, Eliah G. Overbey, Christopher Mason, David Furman

## Abstract

Cover legend: The cover image is based on the article *Astronauts as a Human Aging Model: Epigenetic Age Responses to Space Exposure* by Matías Fuentealba et al., https://doi.org/10.1111/acel.70360.